# Detection and characterization of pathogenic *Pseudomonas aeruginosa* from bovine subclinical mastitis in West Bengal, India

**DOI:** 10.14202/vetworld.2017.738-742

**Published:** 2017-07-04

**Authors:** S. Banerjee, K. Batabyal, S. N. Joardar, D. P. Isore, S. Dey, I. Samanta, T. K. Samanta, S. Murmu

**Affiliations:** 1 Department of Veterinary Microbiology, Faculty of Veterinary and Animal Sciences, West Bengal University of Animal and Fishery Sciences, Kolkata, West Bengal, India; 2 Avian Influenza Laboratory, Institute of Animal Health & Veterinary Biologicals (R&T), Government of West Bengal, Kolkata, West Bengal, India; 3 Department of Veterinary Public Health, Faculty of Veterinary and Animal Sciences, West Bengal University of Animal and Fishery Sciences, Kolkata, West Bengal, India

**Keywords:** bovines, characterization, *exo*S, *Pseudomonas aeruginosa*, subclinical mastitis, *tox*A

## Abstract

**Aim:**

Subclinical mastitis in bovines is mainly responsible for the huge economic loss of the dairy farmers, of which *Pseudomonas aeruginosa* is one of the causative agents. The study was aimed at a screening of suspected milk samples from different cattle farms of West Bengal for detection and confirmation of *P. aeruginosa* strains followed by their characterization.

**Materials and Methods:**

Around 422 milk samples were screened from different dairy farms primarily by on-spot bromothymol blue (BTB) test and then in the lab by somatic cell counts (SCC) to finally consider 352 samples for detection of *P. aeruginosa*. Selective isolation and confirmation of the isolates were done using selective media, *viz*., cetrimide and Pseudomonas agar followed by confirmation by fluorescent technique. Molecular characterization of the strains was done by polymerase chain reaction for the presence of *tox*A (enterotoxin A, 352 bp) and *exo*S (exoenzyme S, 504 bp) genes.

**Results:**

Approximately, 371 (87.9%) samples were positive in on-spot BTB test among which 352 (94.8%) samples revealed high SCC values (more than 3 lakh cells/ml) showing infection when screened. Among these, 23 (6.5%) samples yielded typical *Pseudomonas* sp. isolates out of which only 19 (5.4%) isolates were confirmed to be *P. aeruginosa* which showed characteristic blue-green fluorescence due to the presence of pigment pyoverdin under ultraviolet light. Out of these 19 isolates, 11 isolates were positive for *tox*A, 6 isolates for *exo*S, and 2 for both these pathogenic genes.

**Conclusion:**

Approximately, 5.4% cases of bovine subclinical mastitis infections in South Bengal were associated with *P. aeruginosa* which possess pathogenic genes such as *tox*A (63.2%) and *exo*S (36.8%).

## Introduction

India is one among the largest milk producing countries in the world right now. Dairy industry plays a significant role in livestock economy by generating self-employment. Dairy industry by cooperative society brought socioeconomic transformation in innumerable small, marginal dairy farmers and downtrodden people of mainly western part of India along with other parts the country. These livestock farmers in India mainly contribute the major total national milk production which now rose up to approximately 132.4 million tons in 2012-13 from 17 million tons in 1950-51 [1]. One of the major drawbacks in this industry is a loss of milk production due to mastitis and mastitis related problems of the herd. Subclinical mastitis is one of the major causes of milk production loss in India [2]. An estimated total loss of INR 26460 million due to subclinical mastitis of cows in India was reported by Dua [3]. As it is very difficult diagnose in field level by cattle owners, this may lead to chronic infection resulting into irreparable damage of mammary gland of infected cattle, thus culling or elimination cost is also involved in the list of economic losses. Approximately, 12% loss in milk yield due to subclinical mastitis with an average loss of Rs. 1016/- per subclinical mastitis case was also reported by Patel *et al*. [4]

Studies on the etiological factors of subclinical mastitis revealed that bacteria were the major cause followed by different types of viruses, algae, and mycoplasma [2]. One of the major bacterial pathogens associated with this infection *Pseudomonas aeruginosa* [5]. A study at Jammu and Kashmir, India [5] revealed high positivity of subclinical mastitis in a herd of crossbred cows caused by different pathogens along with *P. aeruginosa* (3.6%). These Gram-negative bacteria may possess several pathogenic factors responsible for their pathogenicity, among which exotoxin A (*tox*A) and exoenzyme S (*exo*S) are two major fatal weapons which remain associated with subclinical mastitis infection [6] in bovines.

Therefore, this study was aimed at the detection and molecular characterization of *P. aeruginosa* from subclinical mastitis cases of bovines from different districts of West Bengal for developing an idea about the significance of this infection.

## Materials and Methods

### Ethical approval

The study was approved by Institutional Biosafety Committee of the University and as per the Committee for the Purpose of Control and Supervision on Experiments on Animals rules; it does not require any approval of Institutional Animal Ethics Committee.

### Sample collection

All milk samples (422 in no.) were screened by on-spot positive bromothymol blue (BTB) test according to the standard method [7] with BTB paper, and 371 samples were collected (aseptically directly from the teats in sterile vials) from different organized government farms (8 farms were covered) and small rural farmers (46 farmers were considered for collection) of different districts of South Bengal for this study during October 2013-April 2014 (i.e., total 7 months which were the time period fixed for this study). The samples were collected from cattle irrespective of age and species, and no specific antibiotics were used as per farm records. All 371 samples were tested somatic cell counts (SCC) study in the lab as per the method described by Sharma *et al*. [8] with slight modification.

Dried smears of suspected milk samples were stained by Newman’s modified stain (HiMedia) followed by counting of somatic cells and tentatively infected samples (n=352) with SCC value above 300,000/ml [9] were collected finally (Table-1) and stored at 4°C for further study.

**Table-1:** Distribution pattern of positive samples in different farms/regions.

Name of the regions	Total collected samples	BTB positive (%)	SCC positive samples with over 3 lakh cells/ml (%)	Average SCC value in lakh cells/ml
Haringhata cattle farms	56	46 (82.1)	43 (93.5)	3.26
Kalyani SLFs	49	41 (83.7)	37 (90.2)	3.54
Borsul area	98	86 (87.7)	81 (94.2)	4.88
Memari area	106	100 (94.3)	97 (97.0)	3.92
Mogra area	113	98 (86.7)	94 (95.9)	4.67
Total	422	371 (87.9)	352 (94.8)	4.05

SCC=Somatic cell counts, BTB=Bromothymol blue

### Isolation and characterization

Tentatively subclinical mastitis infected samples after collection were cultured for detection of desired pathogen. Each milk sample under study was streaked on sterile nutrient agar plates after overnight enrichment followed by overnight incubation at 37°C. Bacterial colonies with green color pigmentation were examined morphologically by Gram’s staining method and all Gram-negative bacilli found were further cultured on sterile cetrimide agar plates for final purification [10]. The greenish single colonies were picked up in sterile cetrimide agar slants for further characterization by different biochemical tests, *viz*., such as oxidase test, catalase test, nitrate reduction, indole test, methyl red test, Voges-Proskauer test, citrate utilization and glucose fermentation test as per Quinn *et al*. [10] and Carter and Wise [11]. All positive samples were tested twice to be sure of the result.

### Confirmation of P. aeruginosa strains

Production of pigments such as pyocyanin and pyoverdin by the purified isolates were tested by “fluorescent technique” after growing the isolates on cetrimide and Pseudomonas agar followed by ultraviolet (UV) illumination [12] for confirmation.

### Detection of pathogenic genes in P. aeruginosa isolates

Pathogenic *P. aeruginosa* isolates were confirmed by detection for enterotoxin A (*tox*A) and exoenzyme S (*exo*S) genes in them. DNA isolation and detection of pathogenic genes in all positive isolates were performed following methods of Lanotte *et al*. [13] and Brenner *et al*. [14]: Young broth culture (at 10 ml) of each isolate was harvested in 15 ml TE buffer (40 mM Tris/HCl, 2 mM EDTA, pH 8.0) and lysed in 220 µl of a 25% (w/v) aqueous solution of sodium dodecyl sulfate (SDS) and 30 µl pronase (an enzyme used for degradation of proteins during isolation of DNA). The mixture was then incubated overnight at 37°C to allow cell lysis. DNA was extracted [14] and was resuspended in 1 ml 1× TE for further use. A standard culture of *P. aeruginosa* (ATCC No. 27853) (as positive control) and one *Escherichia coli* isolate (departmental isolate) (as negative control) were used in this study for confirmation.

Two sets of primers (for *tox*A (352bp) (FP 5’GGTAACCAGCTCAGCCACAT3’, RP 5’ TGATG TCCAGGTCATGCTTC3’) and *exo*S (504bp) genes (FP 5’CTTGAAGGGACTCGACAAGG3’, RP 5’TTCAGGTCCGCGTAGTGAAT3’)) as per Lanotte *et al*. [13] were utilized to amplify the specific gene in thermal cycler under specific polymerase chain reaction (PCR) conditions as follows.

The PCR mixture contained PCR buffer (10 mM Tris/HCl, 50 mM KCl, 1.5 mM MgCl_2_, pH 8.3), 200 µM of each dNTP, 12.5 pmol of each primer, dimethyl sulfoxide at a final concentration of 4%, 1U Ampli *Taq* DNA polymerase and 25 ng DNA template (protocol followed as described by Lanotte *et al*.) [13]. The DNA was amplified in a thermal cycler (Eppendorf, Germany) using the following protocol: 94°C for 3 min, 30 cycles of 94°C for 30 s, 55°C for 1 min and 72°C for 1 min 30 s, and 72°C for 5 min. Each gene was amplified separately. PCR products were separated in a 1% agarose gel for 1 h at 100 V, stained with ethidium bromide and detected by UV transillumination.

### Statistical analysis

All data recorded in this study were statistically analyzed using general linear model of IBM SPSS software (version 20).

## Results

Primary screening of all milk samples by on-spot BTB test revealed that approximately 371 (87.9%) samples were positive out of which samples from Memari (94.3%) and Borsul (87.7%) blocks were highly positive than other farms. The somatic cell study of these samples showed that almost all (94-97%) samples were having high SCC average values of 3.92-4.88 lakh cells/ml (Table-1) which were indicative of infection. Samples with lower SCC values or normal SCC values were not considered for bacterial isolation.

**Figure-1:**
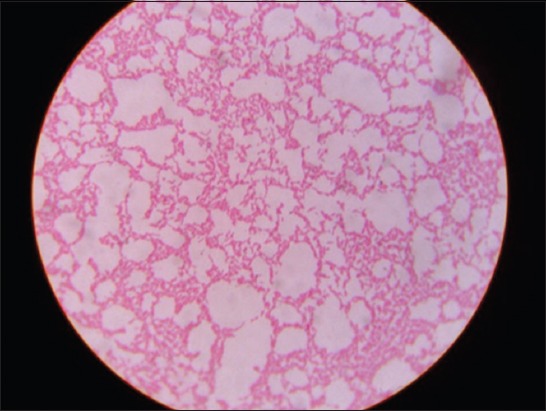
Pink colored bacilli of *Pseudomonas aeruginosa* isolates in Gram’s staining.

On testing for bacterial isolation, a total of 23 (6.5%) samples were found to be positive for *Pseudomonas* sp. as they showed characteristic bluish-green pigmentation on cetrimide agar and were found to be Gram-negative bacilli in nature (Figure-1). Biochemical characterization of these isolates revealed that 21 samples were typical in nature, i.e., positive for oxidase, catalase, citrate utilization, nitrate reduction, and glucose fermentation whereas were negative for methyl red, Voges-Proskauer, and indole tests. Two [2] *Pseudomonas* sp. samples showed variable result in glucose fermentation test as revealed in this study.

**Figure-2:**
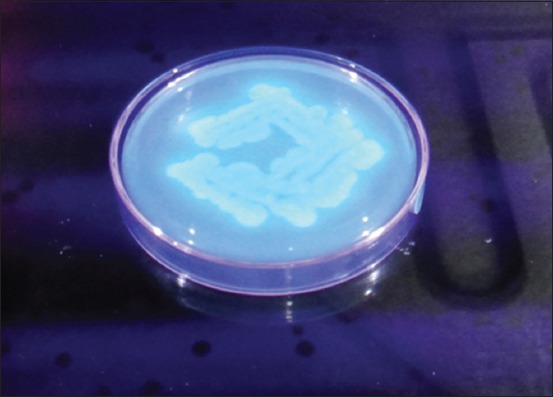
Blue-green fluorescence under ultraviolet light of *Pseudomonas aeruginosa* isolates.

**Figure-3:**
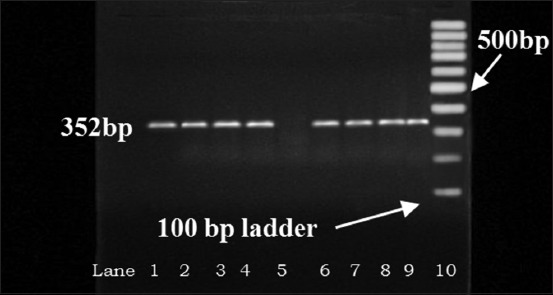
Detection of *tox*A gene (352 bp) in *Pseudomonas aeruginosa* isolates by polymerase chain reaction. Lane 2-4, 6-9: Test samples, 1: Positive control, 5: Negative control.

**Figure-4:**
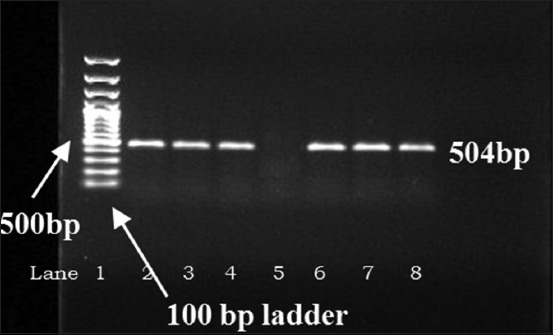
Detection of *exo*S gene (504 bp) in *Pseudomonas aeruginosa* isolates by polymerase chain reaction. Lane 3-4, 6-8: Test samples, 2: Positive control, 5: Negative control.

All 23 samples positive for *Pseudomonas* sp. (including 2 variables) were tested by fluorescent technique to detect 19 (5.4%) isolates showing characteristic blue-green fluorescence due to pigment pyoverdin under UV light (Figure-2) which is confirmatory for *P. aeruginosa*. Samples from Memari block showed highest positivity (7.2%) followed by others (Table-2). Molecular characterization of all 19 positive isolates revealed that 11 isolates to be positive for the presence of *tox*A (352 bp) gene, 6 isolates for *exoS* (504 bp) gene, and 2 isolates to possess both the genes (Table-2, Figures-3 and 4). The presence of *tox*A (63.2%) was found to be much higher than that of *exo*S (36.8%) in *P. aeruginosa* strains as found in thus study.

**Table-2:** Distribution pattern of *Pseudomonas aeruginosa* isolates in different regions with genetic characterization.

Parameters of characterization	Name of the regions					

	Haringhata cattle farms	Kalyani SLFs	Borsul block	Memari area	Mogra area	Total
SCC positive samples	43	37	81	97	94	352
Positive *Pseudomonas* sp. detected	1	2	6	8	6	23
Positive *Pseudomonas aeruginosa* confirmed	1	2	4	7	5	19
Percentage of positivity	2.3	5.4	4.9	7.2	5.3	5.4
*Pseudomonas aeruginosa* with *tox*A	1	1	2	3	4	11
*Pseudomonas aeruginosa* with *exo*S	0	1	2	3	0	6
*Pseudomonas aeruginosa* with both *exo*S+*tox*A	0	0	0	1	1	2

SCC=Somatic cell counts

## Discussion

Approximately, 371 (87.9%) samples were found to positive by on-spot BTB test (which indicates tentative infection) in this study which is more or less matching with the reports of Harini and Sumathi [15], Marschke and Kitchen [16] and Singh *et al*. [17] who also detected approximately 75-90% positivity in their study for subclinical mastitis in bovines. The change in pH of the infected milk samples can be detected by this BTB test, and as the change may be due to other noninfective causes too, that’s why secondary screening with SCC study was performed and the samples also revealed very high average SCC value of these samples (4.05 lakh cells/ml) which were clearly indicative of infection [8], which are also in agreement with the reports of Sharma *et al*. [8] and Smith [9]. Again, Langer *et al*. [18] and Shitandi *et al*. [19] also reported average SCC values of 3-9 lakh cells/ml of milk in their study of bovine subclinical mastitis from Bikaner and Kenya.

A total of 23 (6.5%) samples were found to be typically *Pseudomonas* sp. as per Carter and Wise [11], Quinn *et al*. [10] and Samanta [12]. The slightly variable samples were also previously reported by Freitas and Barth [20] in their studies.

Only 19 (5.4%) isolates were confirmed to be *P. aeruginosa* showing characteristic pyoverdin associated blue-green fluorescence which was also reported by Quinn *et al*. [10], Samanta [12], and Narayanan [6]. Association of *P. aeruginosa* in bovine subclinical mastitis cases were also reported earlier in Gujarat [4] and Jammu and Kashimr [5] at 3.6% and 9.4% respectively, which are more or less matching with this study (5.4%). Other workers such as Heleili *et al*. [21] (3.0%) and Vishwakarma [22] (6.9%) also reported such prevalence of *Pseudomonas* spp. in subclinical mastitis cases of bovines and buffaloes.

The presence of toxic gene like *tox*A (63.2%) was more than that of *exo*S (36.8%) among all 19 positive *P. aeruginosa* isolates were also reported by Lanotte *et al*. [13] who detected *tox*A (approximately 100%) and *exo*S (84.5%) genes in 162 *P. aeruginosa* isolates. The occurrence of “exotoxin A” (90-100%) and “exoenzyme S” (50-84%) in *P. aeruginosa* strains was also reported by Badr *et al*. [23], Nikbin *et al.*, [24] and Sharma *et al*. [25].

## Conclusion

It can be concluded that approximately 5.4% of the subclinical mastitis cases of bovines in different districts of mainly South Bengal, might be caused by pathogenic *P. aeruginosa* strains which possess pathogenic genes, *viz*., *tox*A (63.2%) and *exo*S (36.8%). As this pathogen can act as opportunistic one for the human beings also, so proper care should be taken to check this infection.

## Authors’ Contributions

SB, SM, KB, SD, and DPI prepare the study design and carried out the experiment. SB, TKS, and SD conducted the molecular part of the research. KB, IS, and SNJ analyzed the data, drafted and revised the manuscript. All authors read and approved the final manuscript.

## References

[1] N.D.D.B. Statistics (2013). Milk Production in India. IST; 2013.

[2] Kumar M, Goel P, Sharma A, Kumar A (2009). In: Compendium of 27^th^.

[3] Dua K (2001). Incidence, etiology and estimated economic losses due to mastitis in Punjab and in India - An update. Indian Dairyman.

[4] Patel J.V, Bhingaradia B.V, Patel B.B, Patel S.B, Patel P.B, Vahora S.P (2012). Study on prevalence of mastitis and antibiotic sensitivity of bacterial isolates recovered from crossbred cows of Anand district of Gujarat. Indian J. Dairy Sci.

[5] Singh R, Sharma N, Soodan J.S, Sudhan N.A (2005). Etiology and sensitivity of bacterial isolates from sub-clinical mastitis in cattle from Jammu region. SKUAST J. Res.

[6] Narayanan S, McVey D.S, Kennedy M, Chengappa M.M (2013). Pseudomonas. Veterinary Microbiology.

[7] Chakrabarti A (2007). A Textbook of Preventive Veterinary Medicine.

[8] Sharma N, Singh N.K, Bhadwal M.S (2011). Relationship of somatic cell count and mastitis: An overview. Asian Australas. J. Anim. Sci.

[9] Smith K.L (1996). Standards for Somatic Cells in Milk: Physiological and Regulatory. Mastitis Newsletter, Newsletters of the IDF No. 144.

[10] Quinn P.J, Markey B.K, Leonard F.C, Fitz Patrick E.S, Fanning S, Hartigan P.J (2011). Veterinary Microbiology and Microbial Diseases.

[11] Carter G.R, Wise D.J (2004). Essentials of Veterinary Bacteriology and Mycology.

[12] Samanta I (2013). *Pseudomonas* and *Burkholderia*. In: Veterinary Bacteriology.

[13] Lanotte P, Watt S, Mereghetti L, Dartiguelongue N, Rastegar-Lari A, Goudeau A, Quentin R (2004). Genetic features of *Pseudomonas aeruginosa* isolates from cystic fibrosis patients compared with those of isolates from other origins. J. Med. Microbiol.

[14] Brenner D.J, McWhorter A.C, Knutson J.K, Steigerwalt A.G (1982). *Escherichia vulneris*: A new species of *Enterobacteriaceae* associated with human wounds. J. Clin. Microbiol.

[15] Harini H, Sumathi B.R (2011). Screening of bovine milk samples for sub-clinical mastitis and antibiogram of bacterial isolates. Vet. World.

[16] Marschke R.J, Kitchen B.J (1985). Detection of bovine mastitis by bromothymol blue pH indicator test. J. Dairy Sci.

[17] Singh R, Bansal B.K, Uppal S.K, Malik D.S (2000). Diagnosis of sub-clinical mastitis: A comparative study of different tests. Indian J. Anim. Res.

[18] Langer A, Sharma S, Sharma N.K, Nauriyal D.S (2014). Comparative efficacy of different mastitis markers for diagnosis of sub-clinical mastitis in cows. Int. J. Appl. Sci. Biotechnol.

[19] Shitandi A, Ogollah H, Nanua J.N (2005). Effect of sub-clinical mastitis on milk composition in the Kenyan smallholder dairy herds. Afr. Crop Sci. Conf. Proc.

[20] Freitas A.L, Barth A.L (2004). Typing of *Pseudomonas aeruginosa* from hospitalized patients: A comparison of susceptibility and biochemical profiles with genotype. Braz. J. Med. Biol. Res.

[21] Heleili N, Ayachi A, Melizi M, Kassah A.L, Mamache B (2012). Prevalence of sub-clinical bovine mastitis and the *in vitro* sensitivity of bacterial isolates in Batna governorate, East of Algeria. J. Anim. Sci. Adv.

[22] Viswakarma P (2008). Studies on Prevalence, Diagnosis, Therapy and Control of Mastitis in Buffaloes. M. V. Sc. Thesis.

[23] Badr H.A.R, El-Nagdy M, El-Sabagh A, El-Din A.B (2008). *Pseudomonas aeruginosa* exotoxin aas a virulence factor in burn wound infections. Egypt. J. Med. Microbiol.

[24] Nikbin V.S, Aslani M.M, Sharafi Z, Hashemipour M, Shahcheraghi F, Ebrahimipour G.H (2012). Molecular identification and detection of virulence genes among *Pseudomonas aeruginosa* isolated from different infectious origins. Iran. J. Microbiol.

[25] Sharma S, Kaur R, Yadav V, Harjai K, Joshi K (2004). Contribution of exotoxin A of *Pseudomonas aeruginosa* in acute & chronic renal infection. Jpn. J. Infect. Dis.

